# Observations on the Efficacy of Olive Oil, for Preventing and Curing the Plague

**Published:** 1801-09

**Authors:** 


### *Obſervations on the Efficacy of Olive Oil, for preventing and curing the Plague; by Dr.* SCHEEL.

A Short time ſince, Mr. Baldwin, formerly Engliſh Conſul at Alexandria, diſcovered the great uſe of frictions with olive oil againſt the plague; on which ſubject he publiſhed a work, containing his method and obſervations at large. This book has been tranſlated into German by Dr. Scheel, who has added ſeveral remarks, of which the following is an abſtract.

"The effects of the olive oil, uſed according to the preſcription of Mr. BALDWIN, appear to be the following: 1. It prevents or diminiſhes at leaſt the abſorption of fluids of all kinds, by anointing the whole body, or by its not uniting with water, without the intervention of gum or alkaline ſalt. This circumſtance deſerves to be particularly regarded for explaining the efficacy of the oily frictions in preventing the infection. It is proved by experience and the evidence of facts, that humidity acts a great part in the different contagia, and eſpecially in the plague; for it has been obſerved, that the diſeaſe never penetrates into countries which are perfectly dry, or where the intenſe heat diſperſes all humidity, as in the interior of Africa; and the plague ceaſes in Egypt and Smyrna at the time of the hot and dry ſeaſon. During the time when the dry north-eaſt wind, called *Harmattan*, is blowing on the gold coaſt of Africa, every epidemical diſorder begins to ceaſe, and even the inoculation of the Small-pox is without effect. It is recorded by by MURATORI, that in a very dreadful plague, by which *Venice* was depopulated in the 16th century, the great heat in the famous glafs manufactory of *Murano*, preferved all the workmen that were expofed to it, from the infection of the plague. A certain degree of humidity is likewife required in the fcented exhalations of bodies for being formed and conveyed to the organ of fmell. Every thing befides, that deprives the body of its natural unctuous covering, opens a way to infection, and MURATORI cautions us therefore very much againft wafhing with foap in the time of a plague, becaufe it promotes infection and brings on the moft dangerous confequences.

"2. It prevents the action of atmofpherical air on the body, and confequently the oxydation of oxydible fubftances in the cutaneous veffels, and obftructs likewife the excretion of thofe fubftances which are formed during that procefs. This idea, however, can only be explained according to Dr. Mitchell's theory of the formation of the contagium; and the ufe of the oil confidered in this point of view, can only take place if the principle of the contagium is lodged in the fkin, without circulating in the blood, as in that cafe the oxydation may more probably proceed in the lungs.

"3. It caufes an accumulation of heat in the body, but efpecially in the fkin, by being not fo good a conductor of heat as the atmofpherical air, and likewife by diminifhing perfpiration, which deprives the body of heat.

"4. It produces a *plethora partialis* in the fkin, by the accumulation of ferous humours that are generally excerned by perfpiration, whereby the excitement, particularly of the fkin, increafes; and if this is heightened to a certain degree by the particular ftate of the body, or by other circumftances, a general fweat fupervenes, by which the excitement is again diminifhed, and the equilibrium reftored. This fweat is extremely beneficial, as it expels the poifon out of the body before it produces the difeafe; it is generally very copious, and breaks out at once over the whole body, on which account it proves critical, and is preferable to a fweat excited by any diaphoretic medicine.

"This method of applying the oil deferves, therefore, to be firft ranked amongft the different remedies that have been recommended for the purpofe of preferving the body from infection, and poffeffes the double advantage of being cheap, and requiring very little time and care, and making the daily change of clothes and the fumigations unneceffary, on which account it is particularly calculated for the poorer clafs of people. It may, however, be obferved, that the oily frictions are not able to prevent all poffibility of infection; for though the moft moſt common way in which the contagion is received into the body, is by the ſkin; yet it ſeems very probable, that it may likewiſe penetrate into the body through the lungs, the mouth, and the inteſtinal canal, a circumſtance that is proved by ſeveral obſervations.

"Olive oil is probably not preferable to any other unctuous ſubſtance for the purpoſe of anointing the body; and in countries where it is not to be had ſo eaſily and ſo good, it may be replaced by any other oily ſubſtance. The addition of wax or ſpermaceti will improve it, and prevent its becoming ſo ſoon rancid. The oily covering ought to be waſhed away from time to time. There is no reaſon to fear that the oil may do harm to health, by ſuppreſſing the perſpiration; but though this is diminiſhed by the oily frictions, it ſeems by no means to be wholly ſuppreſſed; and in caſe it ſhould be ſo, that function of the ſkin is eaſily replaced by the lungs, the kidneys, and the inteſtinal canal.

"For explaining the healing powers of oil againſt the plague, when it has already appeared, Dr. Scheel endeavours to prove from the obſervations of different authors, that the plague is more frequently of the ſthenic than of an aſthenic nature. The characteriſtic properties of the *contagium pestis* appear to be the following;

"1. It is an extremely ſtimulant and penetrant poiſon, of certain chemical and phyſical properties, which are ſtill unknown.

"2. The diſeaſe which is produced by it, keeps no certain and regular periods, like the Small-pox.

"3. The poiſon of the plague poſſeſſes a particular tendency of cauſing tumours in the lymphatic and ſalival glands, and in the tela celluloſa of the ſkin, which frequently diſappear again without any ulceration.

"4. The criſis of this diſeaſe is more rarely perfect than in other contagia. It takes place by a copious ſweat, which removes the pyrexia of the plague, and at the ſame time evacuates the poiſon, or the diſeaſe ceaſes by inſenſible perſpiration, or by the ſuppuration of an eruption attending the plague. Dr. S. is, however, inclined to conſider the buboes merely as a ſymptom, but not as a criſis of the plague, becauſe ſometimes they do not appear at all, and ſometimes at the ſtadium cruditatis, and very frequently the diſeaſe terminates ſucceſsfully without them, merely by a copious ſweat.

"5. It does not render the perſons who have experienced the plague incapable of being again affected with the contagion, as is the caſe in the Small-pox, for the ſame perſons are again expofed to infection, even during the fame epidemic, which, however, but rarely happens.

"The infection proceeds in different ways; fometimes the difeafe fuddenly evolves itfelf by a great quantity of poifon having entered the body; but if only a fmall quantity of it is received into the frame, fome time is required before it can be fo much augmented as to be able to produce the difeafe. It likewife depends on the fubject and his particular difpofition, whether the difeafe comes on fooner or later. It has been generally remarked, that it is promoted by a fthenic diathefis, as in this cafe the acceffion of any ftimulus is likely to produce a hyperfthenia and an indirect debility. The contagion alfo is accumulated in the fkin by the fuppreffion of perfpiration; but it acts more powerfully when it is received through the nofe or with the faliva, and in this way immediately communicated to the circulation. There is alfo fome difference in the phenomena of the difeafe depending on the particular nature of the epidemic, which is in fome inftances more or lefs malignant.

"It has not yet been afcertained, whether a particular ftate of the humour influences the infection, and why the contagion affects particularly the inteftinal canal in fome epidemics, and in others the nervous fyftem.

"The difeafe is generally fthenic in the beginning, but becomes afthenic in perfons whofe food is bad, or in whom fear and grief have produced an afthenic diathefis. The plague is always afthenic in its progrefs, even in robuft people, from an indirect afthenia, and in fome inftances the poifon may act fo violently, as almoft immediately to produce an indirect debility, becaufe the previous hyperfthenia is of fo fhort a duration as hardly to be perceived. The difeafe terminates fatally by diarrhœas and hæmorrhagies fupervening. After having premifed thefe few obfervations on the nature of the plague, the action of the oil may be eafily determined, which accordingly appears to confift, 1. In affecting a critical fweat, that evacuates the poifon and abates the pyrexia. 2. In diminifhing, under certain circumftances, too copious fweats, and preventing a debility that arifes from them. The critical fweat that is occafioned by the frictions with olive oil, ought to be carefully promoted and fupported by antimonials, and to which, in cafe of a tendency to diarrhœas, opiates may be very properly added; and when the fweat begins to weaken the patient, broth, with eggs, wine, &c. ought to be adminiftered. It has been obferved, that fleep is extremely dangerous while the fweat lafts; for it feems as if the fyftem required all the natural ftimuli for rendering this fweat critical, and the leaft deficiency of them would produce a contrary effect. It may be fometimes advifable to add turpentine or juniper oil, in order to increafe the ftimulus in an afthenic asthenic diathesis. The vomiting and diarrhœa require the utmost attention in stopping them. The frictions with oil are also very serviceable in other diseases, for the purpose of counteracting contagions, and preventing an immoderate sweat. Mr. BALDWYN relates two instances, where the spots anointed with oil remained free from the eruption in a child that had been inoculated for the Small-pox, &c."

